# Lighting the way: Recent insights into the structure and regulation of phototropin blue light receptors

**DOI:** 10.1016/j.jbc.2021.100594

**Published:** 2021-03-26

**Authors:** Jaynee E. Hart, Kevin H. Gardner

**Affiliations:** 1 Structural Biology Initiative, CUNY Advanced Science Research Center, New York, New York, USA; 2 Department of Chemistry and Biochemistry, City College of New York, New York, USA; 3 PhD Programs in Biochemistry, Chemistry, and Biology, Graduate Center, City University of New York, New York, USA

**Keywords:** phototransduction, protein kinase, signaling, allosteric regulation, optogenetics, protein engineering, AsLOV2, *Avena sativa* phot1 LOV2 domain, AtLOV2, *Arabidopsis thaliana* phot1 LOV2, Bi, bimolecular fluorescence complementation, FAD, flavin adenine dinucleotide, FCLOV, light–oxygen–voltage, FMN, flavin mononucleotide, HDX-MS, hydrogen–deuterium exchange by mass spectrometry, OT, optogenetic tool, PAS, Per-ARNT-Sim, phot(s), phototropin(s), QE, quantum efficiency, TG, transient grating, FTIR, Fourier transform infrared, SAXS, small-angle X-ray scattering

## Abstract

The phototropins (phots) are light-activated kinases that are critical for plant physiology and the many diverse optogenetic tools that they have inspired. Phototropins combine two blue-light-sensing Light–Oxygen–Voltage (LOV) domains (LOV1 and LOV2) and a C-terminal serine/threonine kinase domain, using the LOV domains to control the catalytic activity of the kinase. While much is known about the structure and photochemistry of the light-perceiving LOV domains, particularly in how activation of the LOV2 domain triggers the unfolding of alpha helices that communicate the light signal to the kinase domain, many questions about phot structure and mechanism remain. Recent studies have made progress addressing these questions by utilizing small-angle X-ray scattering (SAXS) and other biophysical approaches to study multidomain phots from *Chlamydomonas* and *Arabidopsis*, leading to models where the domains have an extended linear arrangement, with the regulatory LOV2 domain contacting the kinase domain N-lobe. We discuss this and other advances that have improved structural and mechanistic understanding of phot regulation in this review, along with the challenges that will have to be overcome to obtain high-resolution structural information on these exciting photoreceptors. Such information will be essential to advancing fundamental understanding of plant physiology while enabling engineering efforts at both the whole plant and molecular levels.

The phototropin blue light receptors (phots) are unique proteins that have had an outsized impact in the radically different fields of plant physiology and protein engineering. In the former, they are key regulators of growth and photosynthetic competence in plants. Their structure, combining small light-perceiving domains with a catalytic output domain that they control, has also inspired creative applications of the phot light-sensing mechanism to artificially regulate unrelated proteins with blue light *via* the development of novel genetically encoded optogenetic tools (OTs) (1). Both of these large fields rely on and benefit from accurate information about phototropin regulation and structure: rationally modifying phots can both boost plant growth under low light, while the design and application of OTs rely on detailed knowledge of aspects of phot regulation by blue light.

Phototropins are present in both algae and plants (2). In algae, a single phot regulates photoprotection (3), eyespot formation (4), and reproduction (5). Due to gene duplication (2), higher plants have two phototropin isoforms, phot1 and phot2, which indirectly influence photosynthesis by altering leaf flatness (6, 7) and chloroplast positioning (6, 8), as well as controlling CO_2_ uptake through stomatal opening (9). Though phot function has diverged somewhat between these lineages, the underlying structure and activation mechanism are conserved (10, 11). The model algal phot from *Chlamydomonas reinhardtii* is somewhat more similar to higher plant phot2 isoforms than phot1, bearing 38% protein sequence identity with phot2 from the model flowering plant *Arabidopsis thaliana versus* 35% identity with *A. thaliana* phot1.

At a domain level, phots are composed of two light-perceiving Light, Oxygen, or Voltage-sensing (LOV) domains (named LOV1 and LOV2 (12)), followed by a serine-threonine kinase domain, which is responsible for propagating the light signal within the cell (12, 13) (Fig. 1). It is important to note that several classes of proteins with somewhat similar domain structures have been identified outside of photosynthetic organisms, but it is unlikely they are evolutionarily related to the phots. These include bacterial LOV-HK proteins with LOV domains coupled to histidine kinases (14, 15) and the fungal and mammalian PAS kinase, which utilizes two Per-ARNT-Sim (PAS) domains (which are a superfamily of environmental sensory domains that include LOV domains) to sense metabolic changes in place of the LOV domains present in phots (16). While these proteins all regulate kinase activity by environmental changes sensed by LOV or PAS domains, there are sufficient differences in structure, regulatory details, and origin that we strongly discourage referring to the latter two groups as “phototropin-like” proteins.

**Figure 1:**
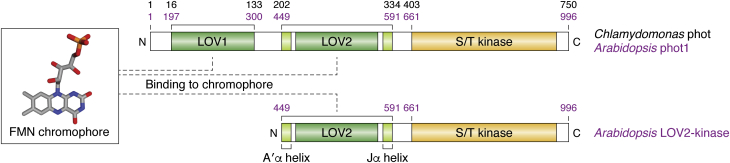
**Phototropin domain composition and nomenclature.** Phots have two N-terminal LOV light-sensing domains followed by a serine/threonine kinase output domain; the LOV2-kinase fragment is an artificially truncated construct of Arabidopsis phot1 that encompasses only the LOV2 and kinase domains. The LOV domains bind an FMN chromophore to enable light perception. LOV2 has two alpha helices, shown in *light green*, which are critical for kinase domain activation. Amino acid numbering and domain boundaries for the *Chlamydomonas* phot are shown in *black*, and for Arabidopsis phot1 in *purple*; note that the LOV2 domain is considered to include both the core LOV domain and the N-terminal A′α and C-terminal Jα helices.

While the function of phot LOV1 domains remains somewhat unclear (see below), extensive biochemical and biophysical work shows that LOV2s repress kinase activity in darkness, which is released by the light-induced disordering of two alpha helices (“A′α” and “Jα”) that flank the LOV2 domain (17, 18, 19, 20). This process triggers autophosphorylation of the kinase domain, which is the final step in potentiating phot signaling (13). Notably, this light-induced protein unfolding event is not only the linchpin in initiating phot activity, but has also been exploited in the design of a collection of OTs, which regulate diverse cellular phenomena (1), including tracking the animal cardiac pacemaker (21), regulating cellular mechanosensing (22), and controlling neuronal networks (23). LOV2-based OTs, while extremely successful on many fronts, are still somewhat limited by the equilibrium between the dark and lit states: there is always residual activity in darkness, and the combination of thermal reversion and inefficiency in allosteric coupling ensures that some molecules spontaneously deactivate in light (18, 24). Improving OTs thus benefits from a detailed understanding of LOV2 light activation, particularly regarding how phot LOV2 domains interact with their adjacent A′α and Jα helices and how, in turn, these interact with the kinase domain.

While much progress has been made in understanding the photochemistry and early light-induced conformational changes of individual LOV domains, we still have an incomplete understanding of important aspects including structures of the full-length phot proteins and mechanisms linking LOV2 helix release to kinase activation. While recent low-resolution studies have made some inroads, extending these to high resolution has been complicated for us and others in the field by practical issues that likely stem from the multidomain/multilinker architecture of phots and the presence of long activation loops within phot kinase domains (*e.g.*, Nakasako *et al*. (25)). In this review, we will highlight what is known about phot structure and activation, identify outstanding questions in the field, and consider the factors that presently challenge obtaining higher-resolution information on full-length phototropins.

## LOV domain structure and activation

**Figure 2:**
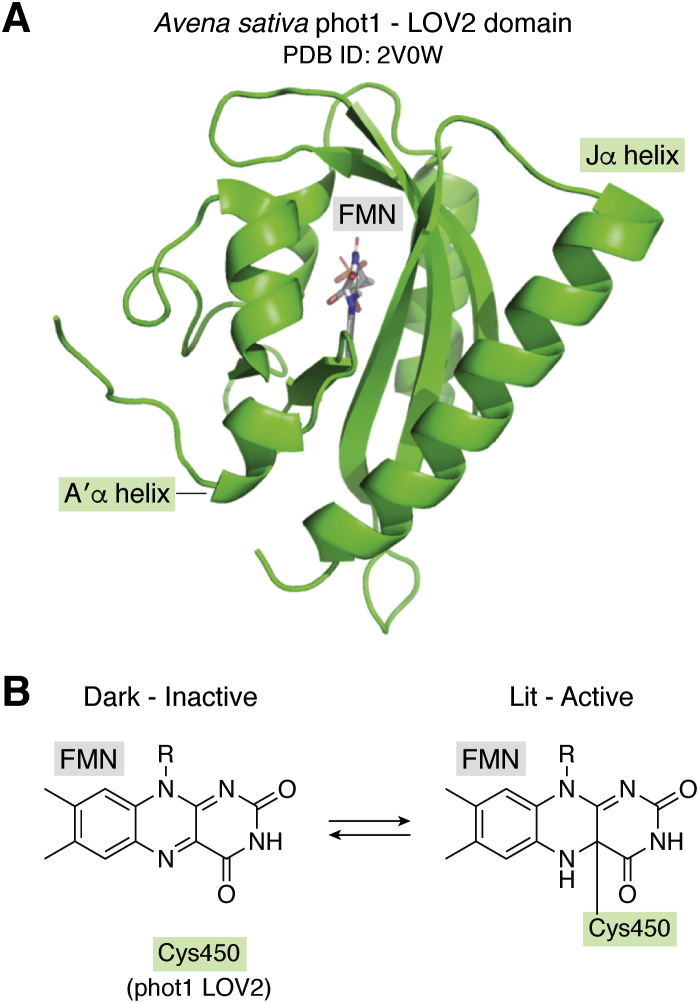
**LOV domain photochemistry and structure.***A*, crystal structure of the LOV2 domain from *Avena sativa* phot1 (PDB ID: 2V0W (90)). *B*, the LOV domain photocycle. Following light stimulus, a covalent bond is formed between FMN and a conserved cysteine within the LOV domain, triggering the activation of the phot.

LOV domains are members of the PAS domain superfamily that specialize in blue light sensing (12, 26, 27). They share the canonical PAS domain fold, composed of a five-stranded antiparallel beta sheet with an extended helical connector linking the second and third beta strands (Fig. 2) (18, 28, 29). Many LOV domains contain important N- and C-terminal helical extensions outside of the minimal domain core, including the aforementioned A′α and Jα helices, which play a critical role in phot LOV2 domains by disordering after light excitation as an integral component of the photoactivation process (18, 19, 20, 30).

In addition to the shared structure of LOV domains, their underlying photochemistry is also well conserved across diverse photoreceptors, including phots (31), and more broadly diverse LOV-containing proteins from bacteria (32), fungi (26), and plants (33). Central to this process is a flavin chromophore, most commonly flavin mononucleotide (FMN) but occasionally flavin adenine dinucleotide (FAD) (34, 35) or riboflavin (36) in certain proteins. While the flavin chromophore is noncovalently bound in darkness, blue light excitation triggers the formation of a covalent photoadduct between the isoalloxazine C4(a) atom and a conserved cysteine residue (C512 in *Arabidopsis* phot1 LOV2 “AtLOV2”; Fig. 2). Concomitantly, the isoalloxazine N5 position becomes protonated, triggering hydrogen bonding changes to an adjacent glutamine residue (Q575) (37, 38). This change is thought to propagate from this glutamine within the flavin-binding pocket to the LOV domain surface; while details of subsequent steps diverge among LOV proteins (1), for phot LOV2 domains this leads to unfolding of the A′α and Jα helices and activation of kinase activity (37, 38, 39). After illumination ends, the cysteinyl photoadduct decays on the timescale of seconds to hours (*e.g.*, t_1/2_=29 s for AsPhot1 (*Avena sativa*) LOV2 at room temperature (33)), returning the domain to its original noncovalent chromophore and folded structural state, thus completing the photocycle that governs the activity of LOV-based photoreceptors.

Interestingly, while the basic characteristics of the photocycle itself are conserved, activated state lifetimes and quantum efficiencies vary substantially among LOV domains in different photoreceptors. Some LOV domains very slowly recover to the dark state: the best-studied example is the fungal photoreceptor VVD, which has a half-lifetime of dark state reversion of 2.5 h (26). Phot LOV2 domains, by contrast, recover to the dark state relatively quickly (33). This fast recovery can limit the light sensitivity and signaling efficiency of both phots and LOV2-based optogenetic tools (1), although this feature also allows such tools to be used in studies of relatively short timescale biological phenomena. Turning to quantum efficiency (QE), phot LOV2 domains tend to have higher QEs than LOV1s although this varies by source: LOV2s from higher plant phot1s are tenfold more efficient than LOV1s (33, 40), dropping to twofold more efficient in the *Chlamydomonas* phot and higher plant phot2s (33). Combined with differences in photocycle length, phot1 dominates phot2 in most responses in higher plants (6, 12, 33). Though united by the same overall structure and mechanism, these differences highlight the functional impact of differences in light sensitivity and quantum efficiency between LOV-containing photoreceptors.

Given this impact on photobiology coupled with engineered applications with OTs, LOV domain photocycles have been extensively studied and modified through random (41, 42) and rational (26, 43, 44) mutagenesis to tune various features for efficient signaling and on/off kinetics in target systems. Mechanistically, slowing the photocycle to prolong the signaling state generally involves either sterically stabilizing the photoadduct or changing the electronic state around the flavin to favor activation (26). While specific mutations are beyond the scope of this review, we highlight the interested reader to studies that have used structure-guided mutagenesis to tune sensitivity in both optogenetic tools (45, 46) and plants (44). In *Arabidopsis*, introducing mutations to tune the phot1 photocycle appeared to increase light sensitivity and plant growth under dim light conditions. However, one of the tested mutations (AtLOV2 V478L) produced a phot1 variant that exhibited autophosphorylation activity *in vivo* but appeared to be unable to propagate the signal downstream of light activation, as its phenotype in transgenic plants mimicked a *phot1phot2* double mutant for most responses, including leaf flattening and phototropism (44). This result, and others in the broader LOV signaling field, underscores the need to evaluate “tuning” mutations by a mix of assays assessing photocycle, structural, and functional properties—ideally in full-length proteins in both *in vitro* and cellular contexts to ensure that mutations introduce only the anticipated changes.

## The LOV1+2 light sensing unit

While many LOV-containing proteins contain only a single LOV domain (31), phots contain a tandem LOV domain motif (LOV1+LOV2) (12, 31, 47). Both LOV domains are required for full light sensitivity *in planta* (48), and though both LOV1 and LOV2 share the same basic structure and photochemistry, the two domains are not interchangeable (49) and appear to serve different roles. Interestingly, the LOV1+LOV2 unit was reported to show some activity when expressed by itself in *Chlamydomonas* (4), suggesting that at least in this setting, the LOV1+LOV2 unit can fulfill some functions in the absence of the kinase domain. In any case, the preservation of both LOV1 and LOV2 in phots across large evolutionary distances suggests that both domains are important for phot function.

As mentioned above, LOV1 and LOV2 seem to have slightly different roles. Crucially, there is presently no experimental validation of helices flanking LOV1 domains analogous to the functionally-critical A′α and Jα helices adjacent to LOV2 domains, and LOV2 alone is necessary and sufficient for activation of the kinase domain (12, 18, 48, 50). Though a “hinge” region that undergoes light-induced conformational change has been suggested to exist in the linker between LOV1 and LOV2, this is presently supported by a combination of low-resolution experimental information (transient grating, TG) (51) and computational simulations (52, 53) without a clear sense of the functional requirement for such a change. As such, the function of LOV1 is not totally understood, though we do know that its presence increases phot light sensitivity relative to a single LOV2 domain (48). This is particularly interesting because phot1 LOV1 has a tenfold lower QE than LOV2 (33, 40) as mentioned above; indeed, it has been suggested that the role of LOV1 in potentiating LOV2 sensitivity may be through physically interacting with LOV2 rather than through its inherent photosensitivity per se (54). Another key difference between the domains is that isolated LOV1 domains tend to have a much higher propensity to dimerize in solution than LOV2s, leading to several literature models that LOV1 may mediate dimerization of full-length phot and/or interactions with other proteins (51, 53, 55, 56, 57). As such, while the exact role that LOV1 plays remains unclear, it is evident that its presence is necessary for phot1 to be responsive to a wide range of light intensities in *Arabidopsis* (48).

Because LOV2 domains most directly control phot kinase activity, they have been the central focus of structural and mechanistic studies, followed by engineering into OTs. In particular, the oat *A. sativa* phot1 LOV2 domains (AsLOV2) have been applied to regulate outputs in wide range of optogenetic tools (1, 58). Such engineered proteins often have a truncated A′α helix and tend to rely exclusively on the unfolding of Jα to transmit photoregulation to effectors that control outputs as diverse as relocalization, modulation of protein–protein interactions, dimerization (45, 59, 60, 61), or deactivation of an output domain through this induced disorder (62). However, while illumination generates a 70-fold change in the dark/light conformational equilibrium of the Jα helix conformation in AsLOV2, this switch is an imperfect one and can limit the degree of activation in optogenetic tools (63). Learning more about this switch, particularly regarding the relationship between LOV2 activation and the physical release of kinase activity in full-length phot1, will be key to resolving some of these issues.

## Kinase structure and activation

While studies investigating how LOV2 activates the phot kinase domain will best be achieved using high-resolution structural biology methods, a combination of currently available biochemical and biophysical studies of *Arabidopsis* phot1 and phot2 (64, 65, 66) suggest that the ∼50 residue linker region between the LOV2 Jα helix and the kinase domain (Fig. 1) may play a key role in kinase activation. In particular, SAXS (small-angle X-ray scattering) data of a LOV2-linker-kinase construct have led to a model where LOV2 sits on top of the kinase domain N-lobe (54, 66, 67). Does the presence of LOV2 somehow perturb the conformation of the kinase N-lobe, preventing activity in darkness that is later relieved after light-induced conformational changes? Or is another mechanism at play? At one point it was believed that LOV2 may occupy the catalytic cleft of the kinase domain (68), though current SAXS-based models of full-length phot structure do not support this (25, 54, 67). More recent studies predict that the linker region between LOV2 and the kinase domain may form two short alpha helices C-terminal to Jα that communicate unfolding to the kinase domain (64, 65). Though this hypothesis is enticing, there has not been any direct evidence of such secondary structure, and the only assay of how mutations in the region affect function was by alteration of kinase activity *in vitro*. More experiments testing this hypothesis will be necessary to elucidate whether this proposed mechanism governs signal transduction to the kinase domain. Key to doing so will be to further explore the structure of the phot kinase domain itself.

While little is directly known about the structure of phot kinase domains, much can be inferred from studies of related mammalian kinases. Phototropin kinase domains belong to the AGC kinase family, a large class containing mammalian, fungal, and plant representatives (69, 70). Typical kinases such as the one from phots have two subdomains: an N-lobe and a C-lobe, separated by the ATP-binding catalytic cleft. Kinases also have an activation loop stemming from the C-lobe and often located between the two lobes, generally present in an unstructured conformation, and containing sites that must be phosphorylated to initiate kinase activity (70). Consistent with this expectation, autophosphorylation at two activation loop residues in the kinase domains of *Arabidopsis* phot1 and phot2 is required for phot function in plants (71, 72). Other common features of the kinase domain include the so-called gatekeeper residue, which allows for specific ATP binding within the catalytic cleft (73). Substitution of this residue with a smaller one enabled *Arabidopsis* phot1 to accommodate bulky ATP analogues using the “bump-and-hole” approach (74) to facilitate a chemical biology route to identify phot1 substrates (73). Taken together, this information indicates that phots most likely have a standard kinase domain such as those found in other AGC kinases.

**Figure 3:**
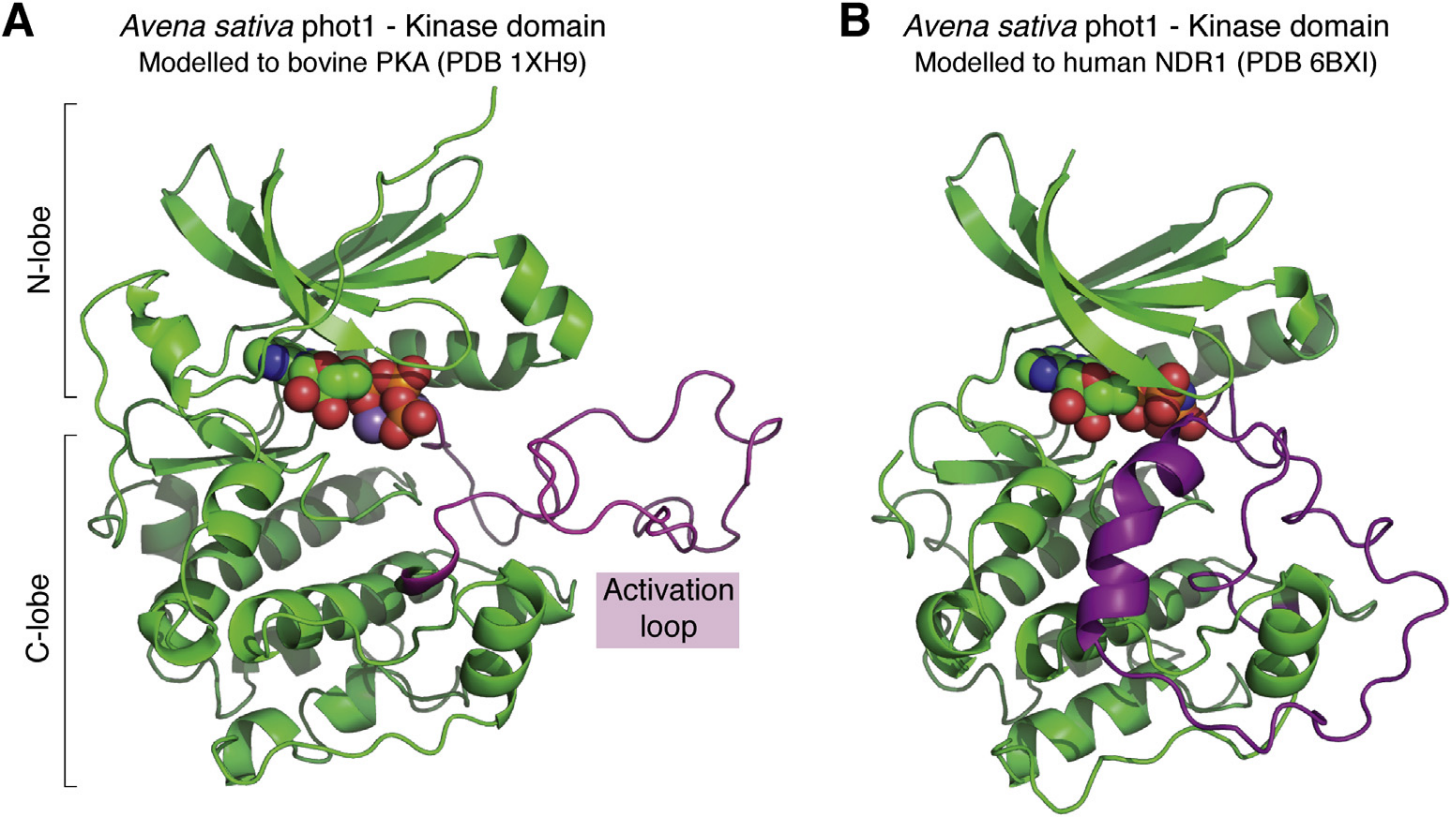
**Models of phototropin kinase domain structure show differential ordering of activation loop depending on template.** The kinase domain of phot1-1 from *Avena sativa* was modeled in-house using Swiss Model (81) with either bovine PKA (*A*; PDB ID: 1XH9 (91)) or human NDR1 (*B*; PDB ID: 6BXI (76)) as the template. Nucleotides are shown in space-filling representation. The activation loop is highlighted in *purple ribbon representation*; note that it does not adopt any stable secondary structure in the PKA-derived model in *panel A*, but partially adopts a helical conformation and is closer to the kinase domain in the NDR1-derived model in *panel B*.

While these common features among kinases seem to hold for phots, one unusual feature complicates structure/function studies: while activation loops are typically ca. 20–30 residues in length (75), these are uncommonly long in phototropins (57 residues in *Arabidopsis* phot1), as for a handful of other AGC kinases (*e.g.*, 63 residues in NDR1/2 (76), 75 residues in LATS1/2 (76), and 575 residues in MASTL/GREATWALL (77, 78)). Modeling studies have suggested that the phot activation loop is unstructured (79), and this is supported by our own secondary structure predictions using JPred (80), likely due to a combination of loop length and sequence. We note that a counter-hypothesis may be suggested by a recent crystal structure of the NDR1 kinase domain (PDB:6BXI (76)) where its long activation loop is ordered and coordinated with the kinase domain to block the catalytic cleft, supporting an autoinhibitory role for the segment in the unphosphorylated state. Intriguingly, when we simply model the oat phot1 kinase domain using the *H. sapiens* NDR1 (unpublished modeling for illustration here), the activation loop is also modeled as ordered (Swiss Model (81), with 32% protein sequence identity and 51% similarity between the Asphot1 and HsNDR1 kinase domains); by contrast, comparably modeling with *B. taurus* PKA, a mammalian AGC kinase with a shorter activation loop, the activation loop is modeled as disordered (30% identity and 45% similarity between the kinase domains of Asphot1 and BtPKA) (Fig. 3). Though no direct evidence of the conformation of the phototropin activation loop exists, Fourier Transform Infrared (FTIR) spectroscopy on the full-length *Chlamydomonas* phot suggested that the activation loop may adopt an alpha helical structure that undergoes major conformational changes upon light exposure (79). Later work from another group contradicted the claim that the activation loop of phototropin undergoes significant conformational change using transient grating (65). However, given the order found for the NDR1 activation loop, it is tempting to speculate that phototropin may not have a totally disordered activation loop and that the length and sequence of the loop may be playing some functional role in phototropin activation. Without more direct information on the structure of the phot kinase domain, any light-induced conformational changes within the domain as well as its interaction with LOV2 will remain a substantial unknown variable.

## Models of full-length phot structure and dimerization

**Figure 4:**
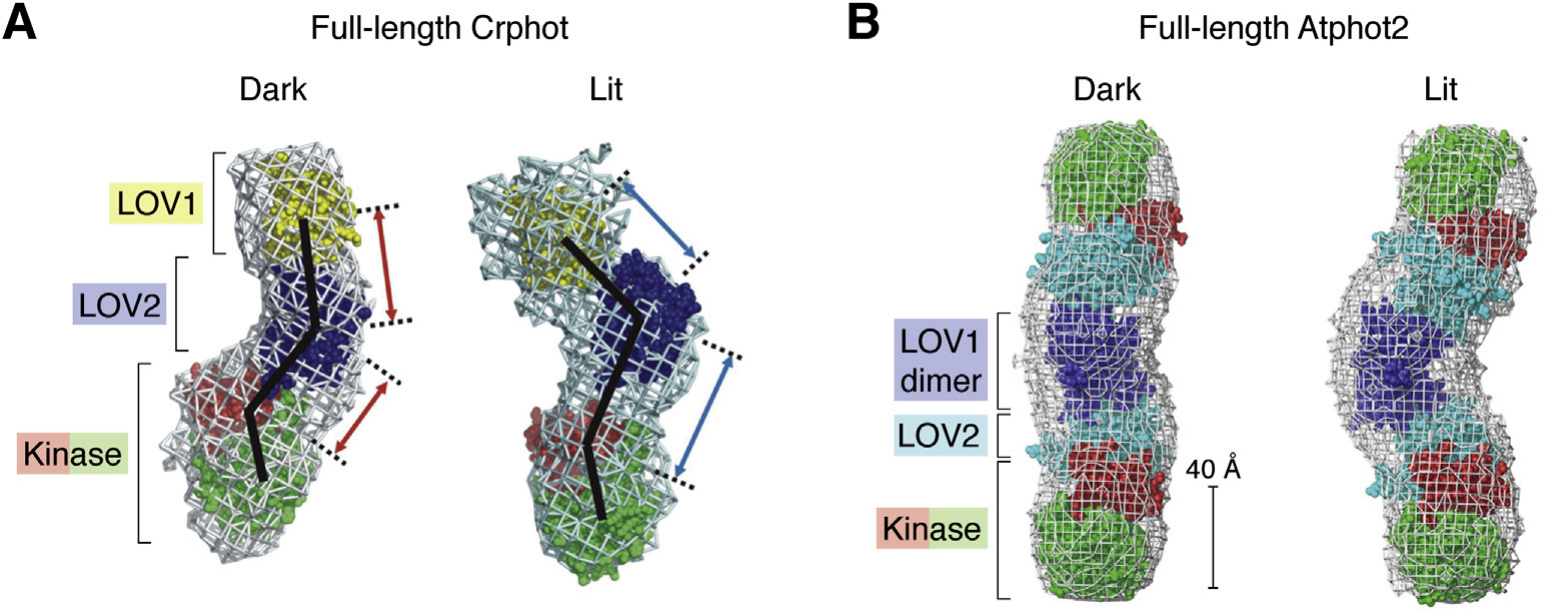
**Current view of phot LOV/kinase interactions based on SAXS analyses of full-length proteins**. *A*, SAXS-derived models of dark and lit Crphot (adapted with permission from Okajima *et al*. (54)). These models depict Crphot as a monomer that extends and inclines in response to light treatment, with particularly noticeable elongation occurring between the centers of mass for the LOV2 and kinase domains (*lower red* and *blue arrows*). In contrast, the LOV1-LOV2 distance has limited light effects (*upper red* and *blue arrows*). *B*, SAXS-derived models of dark and lit Atphot2 (adapted with permission from Oide *et al*. (67)). These models indicate that Atphot2 forms a head-to-head dimer through the LOV1 domains, with illumination converting a relatively linear domain arrangement in the dark into a bent lit-state conformation.

While high-resolution structural studies remain to be reported for multidomain phot fragments—of either LOV2-kinase, which is a model light-regulated phot truncation, or full-length phot protein—several published SAXS studies examining such proteins have usefully contributed to the field over the last few years (54, 66, 67). Studies of the full-length *Chlamydomonas* phot suggest that it is a monomer with an overall shape of a chair, where the kinase domain is the chair's base and LOV1 and LOV2 are the backrest, slightly offset from the legs (54). Upon light activation, the LOV2 domain is modeled to extend substantially away from the kinase domain, accompanied by a rotation of the LOV1-LOV2-kinase angle without adjusting the LOV1-LOV2 distance (Fig. 4*A*) (54). In similar studies of phots from the higher plant *Arabidopsis*, LOV2-kinase from phot1 and full-length phot2, *ab initio* structural modeling from SAXS data supports a head-to-head dimer mediated by LOV1 (in the full-length example) (67) or LOV2 (in LOV2-kinase) (66). Here, illumination seems to convert a relatively linear full-length phot2 dimer into a more bent arrangement, again with a pivot in the LOV1-LOV2-kinase angle (Fig. 4*B*) (66). Significantly, in all models the molecules returned to the dark state structure following illumination (54, 66, 67). While noticeable, the magnitude of changes from the dark state to the lit state is perhaps smaller than that would have been expected from (a) studies on isolated LOV2 domains, which exhibit unfolding of approximately 25 residues in the A′α and Jα helices and (b) anticipated conformational changes in the kinase domain. We note some caveats to this work: the domains are not completely fit within the SAXS-derived scattering envelopes, and these results disagree with a prior literature hypothesis indicating that LOV2 may physically occlude the active site of the kinase domain (30, 68). These points underscore that while present models are useful for consideration at this time, additional experimental evidence, likely *via* a combination of complementary biophysical and structural data plus *in vitro* and cellular functional tests of point mutants, will be needed to fully discriminate among alternative mechanisms of LOV regulation of phot kinase activity.

These structural models obtained by SAXS highlight differences in oligomeric state between the monomeric algal phot from *Chlamydomonas* and the head-to-head dimer observed in phot1 LOV2-kinase and full-length phot2 from the flowering plant *Arabidopsis*. As noted earlier, these phots do not play the same physiological roles. The *Chlamydomonas* phot shares ∼47% protein sequence similarity with *Arabidopsis* phot1, which may be different enough to account for differences in dimerization between them. However, as phots from algae, including *Chlamydomonas* and *Ostreococcus tauri,* are partly or fully functional for many phot-mediated responses in *Arabidopsis* (10, 11), it is not clear how the observed differences in *in vitro* dimerization are functionally important.

From our perspective, the head-to-head dimerization model proposed for the phots from *Arabidopsis* is a surprising finding. Bimolecular Fluorescence Complementation (BiFC) studies conducted *in planta* using *Arabidopsis* phot1 (heterologously expressed in tobacco) reveal light-induced physical association between phot1 molecules at the C-terminus that is independent of kinase activity (49, 82). The differences between the light dependence of dimerization *in planta versus in vitro* by SAXS is particularly striking. However, it is difficult to compare BiFC, which does not provide direct structural information and can capture transient events, to the SAXS model. Another piece of information that is difficult to reconcile with a head-to-head dimer model is that phot1 has been reported to undergo autophosphorylation in *trans* (20, 49), as has been reported for mouse PDK1 (83), another AGC-family kinase. In the SAXS model, the kinase domains are on opposite sides of the dimer: phosphorylation between these molecules in *trans* would likely require a higher-order oligomerization, which was not reported (66, 67). Investigation of dimerization at different concentrations and illumination regimes using a technique such as multiangle light scattering, as well as attempting to rationally identify mutations that could disrupt dimerization, would provide useful information that could help dissect differences between experimental approaches.

## Conclusions and remaining challenges

From our perspective, three key questions remain very open regarding phot structure and regulatory mechanism: the function of LOV1 within the full-length photoreceptor, the structure of the kinase domain, and what light-induced conformational changes it may undergo, and most importantly, how the unfurling of the LOV2 A′α and Jα helices communicates with the kinase domain to induce activation. Progress on any of these questions would improve OT design as well as provide clues as to how to engineer phots in plants for increased productivity. Some of these questions may be answered through continued investigation of fragments of phots, such as using LOV1+2 to probe how those domains relate to one another or LOV2-Kinase to study kinase activation, but full-length structures would provide much more information, such as whether full-length phots undergo light-induced dimerization, as has been observed *in vivo* (49, 82) and in other LOV-containing photoreceptors such as EL222 (84) and Aureochrome1a (85).

At present, several factors appear to be limiting in generating high-resolution information on phots. Some of these issues are practical, particularly in difficulties with expressing and purifying sufficient quantities of intact and functional phototropins for structural interrogation, as observed in published works in the field (25, 54) and supported by anecdotal evidence from our lab and others. This seems to be particularly relevant for phot1 from higher plants, where the best information available is from SAXS on LOV2-kinase fragments (66) rather than the full-length protein. Additional challenges appear to be inherent to the long linkers either between or within various phot domains, including LOV1-LOV2 (145 residues in Atphot1, with little predicted secondary structure), LOV2-kinase (70 residues in Atphot1, with the Jα helix included as roughly 20 residues of this sequence), and the kinase activation loops (57 residues in Atphot1). Finally, the “switchable” nature of the phots necessitates a relatively low stability of regulatory protein/protein interactions to facilitate changes upon illumination.

However, for the field to move forward and obtain mechanistic information on phot activation and its conformational changes, it will be necessary to find ways to obtain a structure or generate a more comprehensive model of the full-length protein. Techniques such as hydrogen–deuterium exchange followed by mass spectrometry (HDX-MS) (86), which has been applied in other LOV photoreceptors to localize light-induced conformational changes to specific regions of the protein (14), could be extremely informative in terms of discovering more about the light activation mechanism, particularly within the understudied phot kinase domain. However, cryogenic electron microscopy (cryo-EM) may be the key technique for bridging the information gap: it requires smaller amounts of protein than many other structural approaches (87), is tolerant of conformational heterogeneity, and has recently become amenable to applying to targets the size of phots (*e.g.*, Atphot1 is 110 kDa as a monomer). This technique has been able to generate mechanistic information on activation in other proteins (88, 89), and ideally would be able to capture dark and lit structures of the phots. Some negative stain images of full-length phot2 were shared in Oide *et al.* (67); while no further structural information emerged from these images, it does highlight the potential of this technique for elucidating a higher-resolution structure of a full-length phot. Combining these advances with the rich biochemical and biophysical history of phototropins, our path ahead appears to be well lit.

## Conflict of interest

The authors declare that they have no conflicts of interest with the contents of this article.

## References

[1] Losi A., Gardner K.H., Möglich A. (2018). Blue-light receptors for optogenetics. Chem. Rev..

[2] Li F.W., Rothfels C.J., Melkonian M., Villarreal J.C., Stevenson D.W., Graham S.W., Wong G.K.S., Mathews S., Pryer K.M. (2015). The origin and evolution of phototropins. Front. Plant Sci..

[3] Petroutsos D., Tokutsu R., Maruyama S., Flori S., Greiner A., Magneschi L., Cusant L., Kottke T., Mittag M., Hegemann P., Finazzi G. (2016). A blue-light photoreceptor mediates the feedback regulation of photosynthesis. Nature.

[4] Trippens J., Greiner A., Schellwat J., Neukam M., Rottmann T., Lu Y., Kateriya S., Hegemann P., Kreimer G. (2012). Phototropin influence on eyespot development and regulation of phototactic behavior in Chlamydomonas reinhardtii. Plant Cell.

[5] Huang K., Beck C.F. (2003). Phototropin is the blue-light receptor that controls multiple steps in the sexual life cycle of the green alga Chlamydomonas reinhardtii. Proc. Natl. Acad. Sci. U. S. A..

[6] Sakai T., Kagawa T., Kasahara M., Swartz T.E., Christie J.M., Briggs W.R., Wada M., Okada K. (2001). Arabidopsis nph1 and npl1: Blue light receptors that mediate both phototropism and chloroplast relocation. Proc. Natl. Acad. Sci. U. S. A..

[7] Sakamoto K., Briggs W.R. (2002). Cellular and subcellular localization of phototropin1. Plant Cell.

[8] Kasahara M., Kagawa T., Oikawa K., Suetsugu N., Miyao M., Wada M. (2002). Chloroplast avoidance movement reduces photodamage in plants. Nature.

[9] Kinoshita T., Doi M., Suetsugu N., Kagawa T., Wada M., Shimazaki K.I. (2001). Phot1 and phot2 mediate blue light regulation of stomatal opening. Nature.

[10] Onodera A., Kong S.G., Doi M., Shimazaki K.I., Christie J., Mochizuki N., Nagatani A. (2005). Phototropin from Chlamydomonas reinhardtii is functional in Arabidopsis thaliana. Plant Cell Physiol..

[11] Sullivan S., Petersen J., Blackwood L., Papanatsiou M., Christie J.M. (2016). Functional characterization of Ostreococcus tauri phototropin. New Phytol..

[12] Christie J.M., Swartz T.E., Bogomolni R.A., Briggs W.R. (2002). Phototropin LOV domains exhibit distinct roles in regulating photoreceptor function. Plant J..

[13] Christie J.M., Reymond P., Powell G.K., Bernasconi P., Raibekas A.A., Liscum E., Briggs W.R. (1998). Arabidopsis NPH1: A flavoprotein with the properties of a photoreceptor for phototropism. Science.

[14] Dikiy I., Edupuganti U.R., Abzalimov R.R., Borbat P.P., Srivastava M., Freed J.H., Gardner K.H. (2019). Insights into histidine kinase activation mechanisms from the monomeric blue light sensor EL346. Proc. Natl. Acad. Sci. U. S. A..

[15] Möglich A., Ayers R.A., Moffat K. (2009). Design and signaling mechanism of light-regulated histidine kinases. J. Mol. Biol..

[16] Rutter J., Michnoff C.H., Harper S.M., Gardner K.H., McKnight S.L. (2001). PAS kinase: An evolutionarily conserved PAS domain-regulated serine/threonine kinase. Proc. Natl. Acad. Sci. U. S. A..

[17] Jones M.A., Feeney K.A., Kelly S.M., Christie J.M. (2007). Mutational analysis of phototropin 1 provides insights into the mechanism underlying LOV2 signal transmission. J. Biol. Chem..

[18] Harper S.M., Neil L.C., Gardner K.H. (2003). Structural basis of a phototropin light switch. Science.

[19] Zayner J.P., Antoniou C., Sosnick T.R. (2012). The amino-terminal helix modulates light-activated conformational changes in AsLOV2. J. Mol. Biol..

[20] Petersen J., Inoue S.I., Kelly S.M., Sullivan S., Kinoshita T., Christie J.M. (2017). Functional characterization of a constitutively active kinase variant of Arabidopsis phototropin 1. J. Biol. Chem..

[21] Arrenberg A.B., Stainier D.Y., Baier H., Huisken J. (2010). Optogenetic control of cardiac function. Science.

[22] Valon L., Marín-Llauradó A., Wyatt T., Charras G., Trepat X. (2017). Optogenetic control of cellular forces and mechanotransduction. Nat. Commun..

[23] Boyden E.S., Zhang F., Bamberg E., Nagel G., Deisseroth K. (2005). Millisecond-timescale, genetically targeted optical control of neural activity. Nat. Neurosci..

[24] Strickland D., Yao X., Gawlak G., Rosen M.K., Gardner K.H., Sosnick T.R. (2010). Rationally improving LOV domain–based photoswitches. Nat. Methods.

[25] Nakasako M., Oide M., Takayama Y., Oroguchi T., Okajima K. (2020). Domain organization in plant blue-light receptor phototropin2 of Arabidopsis thaliana studied by small-angle X-ray scattering. Int. J. Mol. Sci..

[26] Zoltowski B.D., Vaccaro B., Crane B.R. (2009). Mechanism-based tuning of a LOV domain photoreceptor. Nat. Chem. Biol..

[27] Losi A., Polverini E., Quest B., Gärtner W. (2002). First evidence for phototropin-related blue-light receptors in prokaryotes. Biophys. J..

[28] Crosson S., Moffat K. (2001). Structure of a flavin-binding plant photoreceptor domain: Insights into light-mediated signal transduction. Proc. Natl. Acad. Sci. U. S. A..

[29] Halavaty A.S., Moffat K. (2013). Coiled-coil dimerization of the LOV2 domain of the blue-light photoreceptor phototropin 1 from Arabidopsis thaliana. Acta Crystallogr. F Struct. Biol. Commun..

[30] Harper S.M., Christie J.M., Gardner K.H. (2004). Disruption of the LOV-Jα helix interaction activates phototropin kinase activity. Biochemistry.

[31] Glantz S.T., Carpenter E.J., Melkonian M., Gardner K.H., Boyden E.S., Wong G.K.S., Chow B.Y. (2016). Functional and topological diversity of LOV domain photoreceptors. Proc. Natl. Acad. Sci. U. S. A..

[32] Jentzsch K., Wirtz A., Circolone F., Drepper T., Losi A., Gärtner W., Jaeger K.E., Krauss U. (2009). Mutual exchange of kinetic properties by extended mutagenesis in two short LOV domain proteins from Pseudomonas putida. Biochemistry.

[33] Kasahara M., Swartz T.E., Olney M.A., Onodera A., Mochizuki N., Fukuzawa H., Asamizu E., Tabata S., Kanegae H., Takano M., Christie J.M. (2002). Photochemical properties of the flavin mononucleotide-binding domains of the phototropins from Arabidopsis, rice, and Chlamydomonas reinhardtii. Plant Physiol..

[34] Schwerdtfeger C., Linden H. (2003). VIVID is a flavoprotein and serves as a fungal blue light photoreceptor for photoadaptation. EMBO J..

[35] He Q., Cheng P., Yang Y., Wang L., Gardner K.H., Liu Y. (2002). White collar-1, a DNA binding transcription factor and a light sensor. Science.

[36] Rivera-Cancel G., Ko W.H., Tomchick D.R., Correa F., Gardner K.H. (2014). Full-length structure of a monomeric histidine kinase reveals basis for sensory regulation. Proc. Natl. Acad. Sci. U. S. A..

[37] Nozaki D., Iwata T., Ishikawa T., Todo T., Tokutomi S., Kandori H. (2004). Role of Gln1029 in the photoactivation processes of the LOV2 domain in Adiantum phytochrome3. Biochemistry.

[38] Nash A.I., Ko W.H., Harper S.M., Gardner K.H. (2008). A conserved glutamine plays a central role in LOV domain signal transmission and its duration. Biochemistry.

[39] Iuliano J.N., Collado J.T., Gil A.A., Ravindran P.V., Lukacs A., Shin S., Woroniecka H.A., Adamczyk K., Aramini J.M., Edupuganti U.R., Hall C.R. (2020). Unraveling the mechanism of a LOV domain optogenetic sensor: A glutamine lever induces unfolding of the Jα helix. ACS Chem. Biol..

[40] Salomon M., Christie J.M., Knieb E., Lempert U., Briggs W.R. (2000). Photochemical and mutational analysis of the FMN-binding domains of the plant blue light receptor, phototropin. Biochemistry.

[41] Christie J.M., Corchnoy S.B., Swartz T.E., Hokenson M., Han I.S., Briggs W.R., Bogomolni R.A. (2007). Steric interactions stabilize the signaling state of the LOV2 domain of phototropin 1. Biochemistry.

[42] Kawano F., Aono Y., Suzuki H., Sato M. (2013). Fluorescence imaging-based high-throughput screening of fast-and slow-cycling LOV proteins. PLoS One.

[43] Zayner J.P., Sosnick T.R. (2014). Factors that control the chemistry of the LOV domain photocycle. PLoS One.

[44] Hart J.E., Sullivan S., Hermanowicz P., Petersen J., Diaz-Ramos L.A., Hoey D.J., Łabuz J., Christie J.M. (2019). Engineering the phototropin photocycle improves photoreceptor performance and plant biomass production. Proc. Natl. Acad. Sci. U. S. A.

[45] Strickland D., Lin Y., Wagner E., Hope C.M., Zayner J., Antoniou C., Sosnick T.R., Weiss E.L., Glotzer M. (2012). TULIPs: Tunable, light-controlled interacting protein tags for cell biology. Nat. Methods.

[46] Kawano F., Suzuki H., Furuya A., Sato M. (2015). Engineered pairs of distinct photoswitches for optogenetic control of cellular proteins. Nat. Commun..

[47] Huala E., Oeller P.W., Liscum E., Han I.S., Larsen E., Briggs W.R. (1997). Arabidopsis NPH1: A protein kinase with a putative redox-sensing domain. Science.

[48] Sullivan S., Thomson C.E., Lamont D.J., Jones M.A., Christie J.M. (2008). *In vivo* phosphorylation site mapping and functional characterization of Arabidopsis phototropin 1. Mol. Plant.

[49] Kaiserli E., Sullivan S., Jones M.A., Feeney K.A., Christie J.M. (2009). Domain swapping to assess the mechanistic basis of Arabidopsis phototropin 1 receptor kinase activation and endocytosis by blue light. Plant Cell.

[50] Cho H.Y., Tseng T.S., Kaiserli E., Sullivan S., Christie J.M., Briggs W.R. (2007). Physiological roles of the light, oxygen, or voltage domains of phototropin 1 and phototropin 2 in Arabidopsis. Plant Physiol..

[51] Nakasone Y., Ohshima M., Okajima K., Tokutomi S., Terazima M. (2019). Photoreaction dynamics of full-length phototropin from Chlamydomonas reinhardtii. J. Phys. Chem. B.

[52] Peter E., Dick B., Stambolic I., Baeurle S.A. (2014). Exploring the multiscale signaling behavior of phototropin1 from Chlamydomonas reinhardtii using a full-residue space kinetic Monte Carlo molecular dynamics technique. Proteins: Struct. Funct. Bioinformatics.

[53] Henry L., Berntsson O., Panman M.R., Cellini A., Hughes A.J., Kosheleva I., Henning R., Westenhoff S. (2020). New light on the mechanism of phototransduction in phototropin. Biochemistry.

[54] Okajima K., Aihara Y., Takayama Y., Nakajima M., Kashojiya S., Hikima T., Oroguchi T., Kobayashi A., Sekiguchi Y., Yamamoto M., Suzuki T. (2014). Light-induced conformational changes of LOV1 (light oxygen voltage-sensing domain 1) and LOV2 relative to the kinase domain and regulation of kinase activity in Chlamydomonas phototropin. J. Biol. Chem..

[55] Salomon M., Lempert U., Rüdiger W. (2004). Dimerization of the plant photoreceptor phototropin is probably mediated by the LOV1 domain. FEBS Lett..

[56] Katsura H., Zikihara K., Okajima K., Yoshihara S., Tokutomi S. (2009). Oligomeric structure of LOV domains in Arabidopsis phototropin. FEBS Lett..

[57] Nakasako M., Zikihara K., Matsuoka D., Katsura H., Tokutomi S. (2008). Structural basis of the LOV1 dimerization of Arabidopsis phototropins 1 and 2. J. Mol. Biol..

[58] Kolar K., Knobloch C., Stork H., Žnidarič M., Weber W. (2018). OptoBase: A web platform for molecular optogenetics. ACS Synth. Biol..

[59] Guntas G., Hallett R.A., Zimmerman S.P., Williams T., Yumerefendi H., Bear J.E., Kuhlman B. (2015). Engineering an improved light-induced dimer (iLID) for controlling the localization and activity of signaling proteins. Proc. Natl. Acad. Sci. U. S. A..

[60] Gil A.A., Carrasco-López C., Zhu L., Zhao E.M., Ravindran P.T., Wilson M.Z., Goglia A.G., Avalos J.L., Toettcher J.E. (2020). Optogenetic control of protein binding using light-switchable nanobodies. Nat. Commun..

[61] Takano T., Wu M., Nakamuta S., Naoki H., Ishizawa N., Namba T., Watanabe T., Xu C., Hamaguchi T., Yura Y., Amano M. (2017). Discovery of long-range inhibitory signaling to ensure single axon formation. Nat. Commun..

[62] Dagliyan O., Tarnawski M., Chu P.H., Shirvanyants D., Schlichting I., Dokholyan N.V., Hahn K.M. (2016). Engineering extrinsic disorder to control protein activity in living cells. Science.

[63] Yao X., Rosen M.K., Gardner K.H. (2008). Estimation of the available free energy in a LOV2-Jα photoswitch. Nat. Chem. Biol..

[64] Kashojiya S., Yoshihara S., Okajima K., Tokutomi S. (2016). The linker between LOV2-Jα and STK plays an essential role in the kinase activation by blue light in Arabidopsis phototropin1, a plant blue light receptor. FEBS Lett..

[65] Takakado A., Nakasone Y., Okajima K., Tokutomi S., Terazima M. (2017). Light-induced conformational changes of LOV2-kinase and the linker region in Arabidopsis phototropin2. J. Phys. Chem. B.

[66] Oide M., Okajima K., Kashojiya S., Takayama Y., Oroguchi T., Hikima T., Yamamoto M., Nakasako M. (2016). Blue light-excited light-oxygen-voltage-sensing domain 2 (LOV2) triggers a rearrangement of the kinase domain to induce phosphorylation activity in Arabidopsis phototropin1. J. Biol. Chem..

[67] Oide M., Okajima K., Nakagami H., Kato T., Sekiguchi Y., Oroguchi T., Hikima T., Yamamoto M., Nakasako M. (2018). Blue light–excited LOV1 and LOV2 domains cooperatively regulate the kinase activity of full-length phototropin2 from Arabidopsis. J. Biol. Chem..

[68] Tokutomi S., Matsuoka D., Zikihara K. (2008). Molecular structure and regulation of phototropin kinase by blue light. Biochim. Biophys. Acta Proteins Proteomics.

[69] Rademacher E.H., Offringa R. (2012). Evolutionary adaptations of plant AGC kinases: From light signaling to cell polarity regulation. Front. Plant Sci..

[70] Leroux A.E., Schulze J.O., Biondi R.M. (2018). AGC kinases, mechanisms of regulation and innovative drug development. Semin. Cancer Biol..

[71] Inoue S.I., Matsushita T., Tomokiyo Y., Matsumoto M., Nakayama K.I., Kinoshita T., Shimazaki K.I. (2011). Functional analyses of the activation loop of phototropin2 in Arabidopsis. Plant Physiol..

[72] Inoue S.I., Kinoshita T., Matsumoto M., Nakayama K.I., Doi M., Shimazaki K.I. (2008). Blue light-induced autophosphorylation of phototropin is a primary step for signaling. Proc. Natl. Acad. Sci. U. S. A..

[73] Schnabel J., Hombach P., Waksman T., Giuriani G., Petersen J., Christie J.M. (2018). A chemical genetic approach to engineer phototropin kinases for substrate labeling. J. Biol. Chem..

[74] Allen J.J., Li M., Brinkworth C.S., Paulson J.L., Wang D., Hübner A., Chou W.H., Davis R.J., Burlingame A.L., Messing R.O., Katayama C.D. (2007). A semisynthetic epitope for kinase substrates. Nat. Methods.

[75] Modi V., Dunbrack R.L. (2019). Defining a new nomenclature for the structures of active and inactive kinases. Proc. Natl. Acad. Sci. U. S. A..

[76] Xiong S., Lorenzen K., Couzens A.L., Templeton C.M., Rajendran D., Mao D.Y., Juang Y.C., Chiovitti D., Kurinov I., Guettler S., Gingras A.C. (2018). Structural basis for auto-inhibition of the NDR1 kinase domain by an atypically long activation segment. Structure.

[77] Blake-Hodek K.A., Williams B.C., Zhao Y., Castilho P.V., Chen W., Mao Y., Yamamoto T.M., Goldberg M.L. (2012). Determinants for activation of the atypical AGC kinase Greatwall during M phase entry. Mol. Cell Biol..

[78] Ocasio C.A., Rajasekaran M.B., Walker S., Le Grand D., Spencer J., Pearl F.M., Ward S.E., Savic V., Pearl L.H., Hochegger H., Oliver A.W. (2016). A first generation inhibitor of human Greatwall kinase, enabled by structural and functional characterisation of a minimal kinase domain construct. Oncotarget.

[79] Pfeifer A., Mathes T., Lu Y., Hegemann P., Kottke T. (2010). Blue light induces global and localized conformational changes in the kinase domain of full-length phototropin. Biochemistry.

[80] Drozdetskiy A., Cole C., Procter J., Barton G.J. (2015). JPred4: A protein secondary structure prediction server. Nucleic Acids Res..

[81] Waterhouse A., Bertoni M., Bienert S., Studer G., Tauriello G., Gumienny R., Heer F.T., de Beer T.A.P., Rempfer C., Bordoli L., Lepore R. (2018). SWISS-MODEL: Homology modelling of protein structures and complexes. Nucleic Acids Res..

[82] Xue Y., Xing J., Wan Y., Lv X., Fan L., Zhang Y., Song K., Wang L., Wang X., Deng X., Baluška F. (2018). Arabidopsis blue light receptor phototropin 1 undergoes blue light-induced activation in membrane microdomains. Mol. Plant.

[83] Wick M.J., Ramos F.J., Chen H., Quon M.J., Dong L.Q., Liu F. (2003). Mouse 3-phosphoinositide-dependent protein kinase-1 undergoes dimerization and trans-phosphorylation in the activation loop. J. Biol. Chem..

[84] Nash A.I., McNulty R., Shillito M.E., Swartz T.E., Bogomolni R.A., Luecke H., Gardner K.H. (2011). Structural basis of photosensitivity in a bacterial light-oxygen-voltage/helix-turn-helix (LOV-HTH) DNA-binding protein. Proc. Natl. Acad. Sci. U. S. A..

[85] Banerjee A., Herman E., Kottke T., Essen L.O. (2016). Structure of a native-like Aureochrome 1a LOV domain dimer from Phaeodactylum tricornutum. Structure.

[86] Trabjerg E., Nazari Z.E., Rand K.D. (2018). Conformational analysis of complex protein states by hydrogen/deuterium exchange mass spectrometry (HDX-MS): Challenges and emerging solutions. TrAC Trends Anal. Chem..

[87] Bai X.C., McMullan G., Scheres S.H. (2015). How cryo-EM is revolutionizing structural biology. Trends Biochem. Sci..

[88] Loveland A.B., Demo G., Grigorieff N., Korostelev A.A. (2017). Ensemble cryo-EM elucidates the mechanism of translation fidelity. Nature.

[89] Lee C.H., MacKinnon R. (2018). Activation mechanism of a human SK-calmodulin channel complex elucidated by cryo-EM structures. Science.

[90] Halavaty A.S., Moffat K. (2007). N- and C-terminal flanking regions modulate light-induced signal transduction in the LOV2 domain of the blue light sensor phototropin 1 from Avena sativa. Biochemistry.

[91] Breitenlechner C.B., Friebe W.G., Brunet E., Werner G., Graul K., Thomas U., Künkele K.P., Schäfer W., Gassel M., Bossemeyer D., Huber R. (2005). Design and crystal structures of protein kinase B-selective inhibitors in complex with protein kinase A and mutants. J. Med. Chem..

